# Rationale for short course primaquine in Africa to interrupt malaria transmission

**DOI:** 10.1186/1475-2875-11-360

**Published:** 2012-10-30

**Authors:** Alice C Eziefula, Roly Gosling, Jimee Hwang, Michelle S Hsiang, Teun Bousema, Lorenz von Seidlein, Chris Drakeley

**Affiliations:** 1 Malaria Centre, London School of Hygiene & Tropical Medicine, Keppel St, London, WC1E 7HT, UK; 2 Global Health Group, University of California, San Francisco, 50 Beale Street, San Francisco, CA, 94105, USA; 3 Malaria Branch, Centers for Disease Control and Prevention, 1600 Clifton Rd, Atlanta, GA, 30333, USA; 4 Department of Pediatrics, University of California, San Francisco, San Francisco, CA, 94143, USA; 5 Menzies School of Health Research, Casuarina, NT, 0811, Australia

**Keywords:** *Plasmodium falciparum*, Malaria, Primaquine, 8-aminoquinoline, Transmission, Gametocyte, Glucose-6-phosphate dehydrogenase deficiency, G6PD, Africa

## Abstract

Following the recent successes of malaria control in sub-Saharan Africa, the gametocytocidal drug primaquine needs evaluation as a tool to further reduce the transmission of *Plasmodium falciparum* malaria. The drug has scarcely been used in Africa because of concerns about its safety in people with glucose-6-phosphate dehydrogenase (G6PD) deficiency. The evidence base for the use of primaquine as a transmission blocker is limited by a lack of comparable clinical and parasitological endpoints between trials. In March 2012, a group of experts met in London to discuss the existing evidence on the ability of primaquine to block malaria transmission, to define the roadblocks to the use of primaquine in Africa and to develop a roadmap to enable its rapid, safe and effective deployment. The output of this meeting is a strategic plan to optimize trial design to reach desired goals efficiently. The roadmap includes suggestions for a series of phase 1, 2, 3 and 4 studies to address specific hurdles to primaquine’s deployment. These include ex-vivo studies on efficacy, primaquine pharmacokinetics and pharmacodynamics and dose escalation studies for safety in high-risk groups. Phase 3 community trials are proposed, along with Phase 4 studies to evaluate safety, particularly in pregnancy, through pharmacovigilance in areas where primaquine is already deployed. In parallel, efforts need to be made to address issues in drug supply and regulation, to map G6PD deficiency and to support the evaluation of alternative gametocytocidal compounds.

## Background

Current World Health Organization (WHO) guidelines recommend the “addition of a single dose of primaquine (PQ) (0.75 mg/kg) to artemisinin-based combination therapy (ACT) for uncomplicated falciparum malaria as an anti-gametocyte medicine, particularly as a component of a pre-elimination or an elimination programme” [1]. However, unlike recommendations for other anti-malarial treatments this does not come with the supporting statement “*Strong recommendation, high quality evidence”.* This is because there are limited data to suggest that primaquine is safe and efficacious for this use, especially to support regulation and licensure. This is striking given that primaquine has been in the anti-malarial drug arsenal since the 1950s and historical studies strongly suggest that primaquine is highly effective at blocking transmission. Worldwide, 20 countries include primaquine as first-line treatment for *Plasmodium falciparum* in their national policy. None of these countries are in Africa [2].

There are an increasing number of reports of declining transmission intensity in many parts of sub-Saharan Africa, bringing malaria transmission to pre-elimination levels in some countries. There is also increasing recognition that additional strategies aimed specifically at the transmission stages of *P. falciparum* are required both to further reduce transmission and to sustain the gains made by current control efforts. The previously high levels of malaria transmission may be one of the main reasons why primaquine has not been used widely in Africa, with only very frequent delivery of the drug being likely to have any impact on transmission [3]. However, the most likely reasons for the limited use of primaquine in Africa are concerns over safety, given the conservation of the glucose-6-phosphate dehydrogenase (G6PD) deficiency polymorphism in the population.

Using an anti-malarial drug with the goal of interrupting malaria transmission rather than clinical cure necessitates a clearly-defined assessment of safety and efficacy with benefits at the individual level and at the community level being considered. For primaquine, the optimal dose to achieve such endpoints remains undetermined. The recommended 0.75 mg/kg dose is associated with significant haemolysis in some susceptible individuals [4-6], but this dose may well be excessive for the transmission-blocking activity [7]. For the purpose of comparison, doses in this report that are expressed as a milligram per kilogram (mg/kg) equivalent assume an average adult weight of 60 kilograms.

The limited safety data available on single dose primaquine has lead to the requirement of prior testing for G6PD deficiency and pregnancy to avert risk. The necessity for this additional testing has a significant impact on the feasibility, cost effectiveness and the achievable population coverage of large scale primaquine-based interventions. More information on the consequences of single-dose primaquine administration on individuals/ populations with a relevant range of G6PD enzyme activity levels is required urgently if 8-aminoquinolines are to be deployed to interrupt transmission.

### Meeting objectives

With these issues in mind, a meeting of experts was convened to review and discuss existing data on the use of primaquine in Africa for transmission-blocking and to examine the road-blocks that could be overcome to enable and inform its safe use.

Specific objectives of the meeting were to:

1. Identify key road-blocks to deployment of short course primaquine or similar drugs in Africa to reduce transmission of falciparum malaria.

2. Reach consensus on study endpoints so as to maximize comparability between transmission prevention studies.

3. Generate a list of deliverables that will move forward deployment of primaquine in Africa.

## Meeting sessions

### Country program perspectives and potential use for primaquine

Chris Drakeley and Roly Gosling introduced the meeting by providing the current context for the use of primaquine and highlighting the fact that the reductions in malaria transmission that have been described in many sub-Saharan African settings may well be linked to increasing spatial, temporal and even demographic heterogeneity in infections. Spatial targeting of control efforts is likely to make interventions, such as mass drug administration (MDA) more feasible [8]. National malaria control programmes that have seen success in malaria control in the last decade are looking to implement new tools to sustain existing reductions and to further reduce transmission. The question is whether primaquine is one of these tools?

Salhiya Ali described current malaria transmission in Zanzibar, which is characterized by perennial and declining transmission. The sporozoite rate decreased from 4.3 in 2005 to 0% in 2009 and the most recent parasite prevalence was 0.067%. Recent Zanzibar Malaria Control Programme reports suggest that transmission has become highly heterogenous with cases restricted to relatively few weeks per year and to a few localities. Primaquine is not used, but its use could be considered to facilitate further reductions by targeting hot spots, or in treating confirmed clinical cases. The local distribution of gametocytaemia and G6PD deficiency is not known.

In Ethiopia, both *P. falciparum* and *Plasmodium vivax* are endemic and Ashenafi Assefa indicated that the malaria strategy for 2011–2015 includes a plan for elimination by 2020. Primaquine was used in Ethiopia for 25 years up until 1990. Chloroquine (CQ) plus primaquine was first-line treatment for both species. There is no documentation of adverse effects due to primaquine in this period. When sulphadoxine-pyrimethamine (SP) was introduced, it was considered not feasible to administer three drugs, therefore, primaquine was dropped. At present, primaquine is used for radical cure of *P. vivax,* but not for *P. falciparum*. The barriers to using primaquine in Ethiopia include: 1) a lack of documentation of the distribution and clustering of G6PD deficiency (small studies suggest that prevalence is between 1.4 and 6.7% among some minority groups) [9], and 2) uncertainty about the efficacy of primaquine for interrupting transmission of *P. falciparum* in Ethiopia.

Karen Barnes gave a historical perspective of malaria control in South Africa. Previously, the country had high levels of malaria transmission. In 1938, there were 22,000 deaths due to malaria in Kwazulu-Natal. Subsequently, an aggressive approach to malaria control including mapping, malaria surveys, and vector control has reduced the burden considerably but case incidence has remained at a steady state since 2001. The Ministry of Health has now set a goal for elimination by 2018. The biggest challenges include imported malaria, and the perception that malaria is not a public health problem, leading to central budget cuts. Given the already aggressive measures in place, the addition of a transmission-blocking drug such as primaquine could be required to achieve elimination. One challenge is that primaquine is only available on an individual patient basis for radical cure of *P. vivax.* In South Africa, the very high rate of tuberculosis and HIV infection means that the potential for drug interactions with other anti-infective therapies must be considered if primaquine is to be used at a population level. The risk of primaquine-associated haemolysis in people living with HIV infection may differ from that in uninfected people.

In contrast to the aforementioned countries, Diadier Diallo reported that malaria transmission in Burkina Faso is still high. The use of a combination of interventions, such as long-lasting insecticidal nets (LLINs), indoor residual spraying (IRS), and effective artemisinin combination therapy (ACT) with a long half-life partner drug such as dihydroartemisinin-piperaquine is a proposed strategy. Co-administration of ACT with primaquine (or alternatives such as methylene blue) for confirmed malaria episodes and mass drug administration (MDA) may help to further reduce transmission. This strategy may be particularly appropriate in the Sahel area where transmission is highly seasonal and relatively low, making it a potential target for elimination activities. Challenges include the high mobility of human and vector populations particularly from Mali and Niger.

### Historical studies on single dose or short course primaquine for blocking transmission of *P. falciparum*

Chi Eziefula highlighted that the current recommendations for primaquine are based on studies with very small numbers of participants. The parent 8-aminoquinoline, pamaquine (or plasmoquine), developed in the 1920s, was shown to have activity against *P. vivax* and *Plasmodium ovale* relapses, and against both sporozoites and gametocytes of all species [10,11]. A derivative of pamaquine, primaquine was developed in the 1940s by the United States army to prevent relapse of *P. vivax* in soldiers returning from Korea and to prevent the import of malaria into the country [12].

In 1973, the WHO recommended a single dose of primaquine (0.75 mg/kg ) for malaria transmission-blocking and considered prior screening for G6PD deficiency unnecessary [13]. It was not until 2010 that the WHO Malaria Treatment Guidelines (Second Edition) changed to indicate that the risks of haemolysis in G6PD deficient patients should be given consideration prior to primaquine-based interventions.

The currently recommended single dose of primaquine is based on limited efficacy data. In 1961, in Liberia, Burgess and Bray found that a single dose of 0.75-1.5 mg/kg primaquine administered to12 children cleared circulating gametocytes by day 9 [7]. In 1961, also in Liberia, Gunders administered 0.45-1.1 mg/kg of primaquine in combination with pyrimethamine to 22 children and adults. Gametocytes were cleared after a mean of 5 days post treatment, and no mosquito infections occurred in feeding assays [14]. Primaquine was paired with amodiaquine (AQ) in a large scale MDA conducted by Clyde in 1962 in a hyperendemic area of Tanzania. More than 15,000 subjects were studied in three clusters: weekly administration, fortnightly administration, and monthly administration. Outcome measures included asexual parasite, gametocyte and sporozoite rates. After six months there was a ten-fold reduction in parasite prevalence with weekly and fortnightly administration but not with monthly administration [3]. Except for the work by Clyde, there are no substantial field data that indicate that single dose primaquine decreases transmission of *P. falciparum*.

Safety data for primaquine use in Africa or African Americans are equally limited despite the fact that they inform contemporary guidelines. Burgess and Bray comment that primaquine was “well-tolerated”[7]. Clyde reported no safety data and it is unclear who was excluded from treatment [3]. In a series of studies in G6PD deficient African-American volunteers, Alving and colleagues showed that, in three individuals, haemolysis occurred with daily administration of 30mg (approximately 0.5 mg/kg) of primaquine. But, after three weeks, the haematocrit recovered and lower doses resulted in less haemolysis. Eight weekly doses of 60 mg and 45 mg were not associated with haemolysis [15,16]. Daily administration of 30mg of primaquine to African Americans resulted in significant haemolysis in 1%, compared to no severe haemolysis when 15 mg was administered [17]. Tolerance in a pregnant woman (28 weeks gestation) has only been reported by Burgess and Bray, but there was no documentation of birth outcomes [7]. In a more recent study, Kenyan school children were randomized to receive 15mg primaquine daily or three times a week as a malaria prophylactic. It is not clear whether G6PD deficient individuals were included and haemoglobin levels are not reported but again the authors note simply that “primaquine was remarkably well tolerated in our studies” [18].

Kevin Baird remarked that any discussion about primaquine efficacy is necessarily also a discussion about toxicity as there are inherent risks of the drug in situations when the individual patient may not benefit. He highlighted the importance of employing the ethical principles of autonomy, justice and beneficence to gametocytocidal therapy [19]. The 45 mg dose of primaquine is based on data obtained in very few, healthy individuals. This dose was proposed in an era where the goal of the US military was not to find the lowest efficacious dose, but rather to show that the drug worked. The first dose-finding study by Alving in 1960 included one single patient [16]. It was subsequently observed that daily but not weekly administration of 0.25 mg/lb of body weight (~0.55 mg/kg) to G6PD deficient-children resulted in haemolysis [20]. Rieckmann and Burgess both showed declines in gametocytes, oocysts and sporozoites following a dose of 45 mg of primaquine but a similar efficacy was seen with lower doses of 30 mg and 15 mg [7,21,22]. Importantly, these evaluations were conducted without co-administration of a blood schizontocidal drug.

In 1944, the US government abandoned pamaquine as a means of preventing relapses of *P. vivax* due to its haemolytic toxicity and drug interactions. Primaquine was introduced as a gametocytocidal agent at the 45mg dose based on Alving’s work, a dose which was readily available and in use for chemoprophylaxis in American soldiers in Southeast Asia at the time. Some significant haemolysis was seen, mostly in African Americans; there were no deaths but there were several cases of renal failure with daily dosing for 14 days[17]. Summarily, the recommended 45 mg dose may be too dangerous for use in mass drug administration, especially given the limited data on transmission reduction with this strategy.

### Recent studies on the use of primaquine in Africa

Data from two Tanzanian studies which employed single dose primaquine were reviewed by Teun Bousema. In the first study, treatment with sulphadoxine-pyrimethamine (SP) and artesunate (As) was given to children aged 3 to 15 years with uncomplicated falciparum malaria. They were randomized to receive placebo or a single dose of 0.75 mg/kg of primaquine on the third day of treatment (day 2). Compared to the control arm, primaquine administration on day 2 decreased the area under the curve of gametocyte density over time and the duration of gametocyte carriage. The effect was apparent for two weeks; using quantitative real time nucleic acid sequence-based amplification (QT NASBA), 3.9% had gametocytes on day 14 in the primaquine arm, and the density was extremely low, compared to a prevalence of 62.7% in the control arm [23]. Haemoglobin fell in both arms but the drop was more pronounced in the primaquine arm. However, this effect was transient and there was no symptomatic anemia. A haemolytic effect was seen even in some individuals without genotypic (A- variant) G6PD deficiency [23].

In a subsequent cluster randomized study, using MDA in lower Moshi [24], single dose primaquine was given with SP plus As treatment to 1110 individuals older than 1 year with primaquine dosages based on weight (approximately 0.75 mg/kg). It was not possible to assess post-intervention incidence or prevalence because *P. falciparum* transmission had dropped to very low levels. However, safety outcomes, based on haemolysis, were available. Moderate haemolysis occurred following primaquine treatment in 40% of G6PD deficient (A- genotype) individuals but in only 4.5% of non-deficient individuals. There was no clinical compromise due to anemia in any of the children, except in one child in the primaquine arm, whose haemoglobin dropped from 8.3 g/dL to 4.8 g/dL. It was noted that in all cases haemolysis was transient, recovering by day 14 after treatment.

As a former colleague of Professor Li Guoqiao, Keith Arnold represented him and presented data from an MDA campaign in Moheli Island, Comoros. Dr. Arnold began by reviewing Professor Li’s work on primaquine in South East Asia, which served as the basis for the drug regimen used in Comoros. In the late 1990s, Professor Li developed CV8 (320 mg piperaquine phosphate, 32 mg dihydroartemisinin, 5 mg primaquine phosphate, 90 mg trimethoprim). An estimated 1.3 million doses of this drug were administered across Vietnam as part of the National Malaria Control Programme in 2000. There were no documented reports of haemolysis. Data were presented from subsequent dose-finding studies. Artequick (dihydroartemisinin piperaquine given at 0 and 24 hours) was administered in clinical cases followed as inpatients for 30 days followed by administration of 6 mg (7 patients), 7.5 mg (3 patients) or 8 mg (32 patients) of primaquine. A 7.5 mg dose of primaquine rendered gametocytes non infectious at 24 hours. Following 8 mg of primaquine, there were oocysts but no sporozoites in membrane-fed mosquitoes. He decided on the use of Artequick + 9 mg primaquine for MDA after performing safety studies using 8 mg and 10 mg doses in small numbers of individuals with G6PD deficiency in South East Asia. An MDA campaign in 2003 in Cambodia using this regimen resulted in a large reduction in population parasite carriage over three years [25].

In Moheli Island, Comoros the baseline *P. falciparum* parasite prevalence in children ranged from 10-95% in 25 villages. Given a mosquito life expectancy of 30 days, the strategy was to give Artequick for three days plus 9 mg of primaquine on day 1 (Round 1) and day 35 (Round 2). Also, beginning on day 21, 9 mg primaquine alone was given every 10 days, 12 times. Patients less than six months of age were excluded. Treatment coverage for both rounds was reported as >90% and data from monitoring between 2007 and 2009 suggested a reduction of parasite prevalence to <5%. The exception was an area on the south of the island where parasite rates decreased from 94% to 19% with frequent migration from a nearby island suggested as the reason for the persistence of parasites. There were no reports of haemolysis, although it was not measured objectively. The baseline prevalence of G6PD deficiency was estimated to be 15%.

### G6PD deficiency prevalence testing and safety issues

G6PD is an essential erythrocytic enzyme. G6PD deficiency is one of world’s most common genetic polymorphisms. Dennis Shanks described the current array of diagnostic tests available to test for G6PD deficiency. Testing of the enzymatic activity of G6PD on freshly-collected blood samples is the most widely used method. The NADPH fluorescent spot test is most commonly used and is currently recommended by the International Committee for Standardization in Haematology, but it requires a UV lamp and is difficult to do on high volumes of samples. Other diagnostic tests include cytochemical assays, DNA sequence analysis of the G6PD gene, and some rapid diagnostic test formats not yet validated for public health application. In theory, testing for G6PD deficiency is not difficult, but most tests have limitations for large-scale field application, such as expense, requirement for electricity, duration of test procedure, and sensitivity of reagents to light and heat, low detection threshold, and relatively low throughput capacity.

Rosalind Howes described G6PD deficiency as being widespread in tropical regions of sub-Saharan Africa, commonly affecting over 15% of the male population, and in some isolated areas of West and Central Africa reaching up to 30% of the male population. It is considered that severe G6PD deficiency is likely to exist in Africa but its prevalence is unknown. Shanks noted that country-wide MDA with primaquine has been used in China and Nicaragua, both areas with a low prevalence of G6PD deficiency and that in both programmes there were some cases of severe haemolysis. The three primary safety/ tolerability issues with primaquine are gastrointestinal upset, methaemoglobinaemia, and haemolytic anemia in those who are G6PD deficient. G6PD enzyme activity is at best a partial biomarker of clinical effect and the clinical effect is likely dependent on other factors including red blood cell count, gender, and other genetic factors.

### Testing for G6PD deficiency

Gonzalo Domingo observed that genotyping for G6PD deficiency is most commonly carried out for known prevalent mutations at the risk of misclassifying study participants with unknown G6PD deficiency traits as normals. Phenotyping, either quantitative or qualitative, determines G6PD activity in red blood cells and can be defined as a relative deficiency in activity compared to a predefined “normal” activity or in absolute terms in units per gram of haemoglobin. Most studies in Africa have used a semi-quantitative/qualitative fluorescent spot test and observed a high degree of discordance between phenotyping and genotyping not limited to just heterozygous women. Other phenotypic tests e.g. cytochemistry can identify heterozygous females. Spectrophotometry is the gold standard and fluorescent spot tests are useful for screening. The ideal specification for a G6PD deficiency test is difficult to achieve as there is no defined acceptable cut-off of G6PD activity. The challenges are that the measurement of enzyme activity is extremely sensitive to temperature, specimen volume, and possibly specimen type. Of the available tests that run on point-of-care platforms, BinaxNOW is limited by its operating temperature and Access Bio by its small sample volume, which may be a source for performance variability. The BinaxNOW test detects a cut-off of 30-40% enzyme activity and was designed to detect hemizygous males. Detecting heterozygous females require platforms that can detect and enumerate intra-erythrocytic G6PD activity. The next steps include an evaluation of currently available tests for G6PD deficiency under ideal laboratory conditions, field evaluation under controlled conditions, and engaging with the diagnostic sector to define a value proposition for point-of-care G6PD deficiency tests. Ongoing efficacy studies for primaquine represent an opportunity to obtain G6PD deficiency cut-off levels.

### Examples of possible study designs— clinical and field-based

Lorenz von Seidlein and Teun Bousema considered the sequence of studies required to establish the role of primaquine in the response to artemisinin resistance as well as for the elimination of falciparum malaria. Before population-level interventions are considered, three main questions will need to be addressed: 1) What drug concentration is needed to inhibit gametocytes, 2) which primaquine regimen is required to achieve these gametocyte inhibitory concentrations and 3) can this dose be safely administered to both sexes and all age groups? Excluding young children and women of reproductive age from MDA will seriously reduce coverage and is likely to render any intervention meaningless. Since a prospective study of giving single dose primaquine during pregnancy is not likely to be approved, retrospective approaches e.g. pharmacovigilance during large field trials should be explored as a way of gaining information about the safety of primaquine in pregnancy.

One option for field evaluation is the cluster randomized trial. A double-blinded community-randomized, placebo-controlled trial in The Gambia evaluated MDA with sulphadoxine- pyrimethamine (SP) plus single dose artesunate (AS1) in 18 villages and achieved 89% coverage [26]. There was an initial decrease in malaria incidence but the effect quickly disappeared. Possible reasons for a failure to reduce transmission intensity might be that the baseline transmission intensity was too high, that there was migration of infected individuals or mosquitoes, or that the drug regimen was not ideal. A double-blinded community-randomized, placebo-controlled trial was conducted in Tanzania in a setting of very low and seasonal malaria transmission (entomological inoculation rate of approximately 2) using MDA with SP on day 1 plus artesunate for 3 days and primaquine on day 3 [27]. Coverage of 93% was achieved, but the study failed to show a reduction in transmission intensity due to the small number of outcome events (*P. falciparum* infections) in both the intervention and control groups. These studies raise two important questions: 1) Are sub-microscopic parasite densities sufficient to sustain transmission and 2) what is the ideal transmission intensity at which to conduct MDA? It was considered that studies designed to detect the community benefit of ACT *versus* ACT plus primaquine would potentially necessitate very large sample sizes and alternative strategies to evaluate MDA should also be considered. The community effect of insecticide-treated bed nets extends beyond the households that use nets and has been estimated by measuring the distance between control and intervention villages and compounds where protection is seen. Such an effect may exist for primaquine- based interventions such that targeted coverage has a high impact. Less ambitious trial designs could encompass treatment of clinical malaria cases, focal screen–and-treat campaigns, or primaquine could be incorporated into active case detection activities using standardized outcome measures such as entomological parameters, gametocyte prevalence by molecular methods, parasite prevalence/ molecular force of infection, and malaria incidence during follow-up.

The design of trials of MDA with primaquine should help inform a potential strategy for interventions. What is the threshold endemicity level at which MDA with primaquine should be considered? How many rounds of MDA are required and at what interval to give a given effect? Even if efficacy and safety can be established, the issue of willingness to participate in MDA must be considered. In settings of very low transmission and minimal risk, e.g. Swaziland, the community might not be as accepting of MDA as compared to a country with higher endemicity as the perceived benefit is lower.

### Potential study endpoints-clinical and field studies

Heiner Grueninger emphasized that study endpoints should be designed to facilitate both effective treatment and increased knowledge of the study drug. In the context of using primaquine for a new indication of transmission-blocking, the study design should address the requirements set by authorities for obtaining regulatory approval to use the drug. Consequently, endpoints should be considered with input from both industry and policy makers in order to expedite drug deployment in endemic settings.

Chris Drakeley discussed biomedical efficacy endpoints. Abrogation of infection in mosquito infectivity studies is a compelling functional bioassay yet only one existing study involving primaquine satisfied Cochrane review criteria (Graves and Gelband, in press). In this study, mefloquine and SP plus primaquine stopped infection over 14 days post treatment [28]. The mosquito feeding assay methods for assessing post treatment infectivity of subjects offer different options for evaluation, but are not standardized. Direct skin feeding of mosquitoes on treated individuals is most representative of natural infection dynamics but presents logistical and ethical concerns. Using venous blood allows both direct membrane feeding but also serum replacement with untreated or treated serum to examine the effect of different serum compositions, such as drug metabolites. Reproducibility of results is an important issue with no clear guidelines on how to feed mosquitoes, how many mosquitoes should be fed per assay, because the robustness of the estimate of prevalence of infection depends on the number fed [29], and on which day post-treatment participants should be tested for infectivity. For example, primaquine has a short half-life so infectivity could be measured after 24 hours, whereas, for the purpose of MDA, it is probably pertinent to know for how long the subject has reduced infectivity and testing for infectivity up to 28 days may be relevant. This latter point could be addressed by staggering sampling time points between participants to reduce the number of bleeds per individual. Further studies may be required to confirm the effect on infectiousness to wild mosquito populations as natural infections have been shown to be successful at very low gametocyte densities suggesting high vector susceptibility[30]. Such feeding experiments may not be warranted or practical for larger field evaluations and a surrogate marker for transmission would be preferable.

Although, there is no standardized, validated marker of infectiousness of the human host, the most widely used marker to compare drugs is the prevalence of gametocytes 7 days post treatment [31]. Gametocyte density is less relevant at low gametocyte counts found in chronic and asymptomatic infections as the correlation between infectivity and low gametocyte density is poor. The measurement of gametocyte prevalence and density depends on the method of detection with 5- to 10- fold differences seen with molecular methods compared to microscopy[32]. Gametocyte densities can be integrated using area under the curve (AUC) to provide an estimate of gametocyte carriage [33,34]. In natural infections this is likely to vary by age with young children with clinical disease having short, intense gametocytaemia (abrogated by drugs or gametocyte death) and older semi-immune individuals, who can have asymptomatic infections for up to a year [35] and maybe longer, with a more prolonged AUC.

The issue of how to tailor the design of studies using primaquine to include endpoints that are meaningful to regulatory authorities was tackled by Justin Green. The key question is what level of evidence do we require in order to use primaquine as a transmission-blocking agent? He referred to ongoing studies using tafenoquine to highlight how bespoke endpoints are being used to achieve licensure. Tafenoquine is an 8-aminoquinoline developed by the US army and the Walter Reed Army Institute of Research (WRAIR) with GlaxoSmithKline (GSK). It has a long half-life (14–17 days), which may confer advantages as an anti-parasitic agent, but also risks, given that the duration of haemolysis in individuals with G6PD deficiency is also prolonged [36]. The drug is slowly metabolized and the parent compound is responsible for the anti-malarial effect [37]. Tafenoquine is being developed as a radical cure of *P. vivax* infection. Green described a dose-ranging study in individuals over 16 years with *P. vivax* infection evaluating chloroquine alone compared with standard dose chloroquine plus primaquine 15mg (for 14 days) and different single doses of tafenoquine (50mg, 100mg, 300mg, and 600mg) given on day 1 or day 2 (NCT01376167). The primary endpoint is relapse at 6 months with secondary endpoints of relapse at 4 months, time to relapse, parasite clearance time, fever clearance time, gametocyte clearance time (by microscopy), safety and pharmacokinetics/ pharmacodynamics.

These pivotal endpoints are designed with regulatory requirements in mind so that wording related to endpoints can be incorporated into a label claim. From the perspective of industry, this can determine the potential volume of sales (the percentage of the primaquine market obtainable). For trials with primaquine, or other transmission-blocking candidates, it is necessary to decide how important it is that the study endpoint is on the label and whether stakeholders demand a “label claim” or an indication for approval. For transmission markers to stand as endpoints for a regulatory level trial, one would need validation that the marker, e.g., a molecular method such as detection of pfs25 with QT NASBA [38] or microscopic gametocytaemia, correlates with transmission.

Typically, the pharmaceutical industry focuses on the risk-benefit of a particular drug in the individual. For primaquine, the drug may be of more benefit to someone other than the recipient, raising the ethical question of whether it is acceptable to give a drug for community benefit. This issue is also pertinent to transmission-blocking vaccines [39]. Justin Green considered that it is crucial that primaquine trials include individuals with G6PD deficiency (including heterozygote females) and describe the risk of haemolysis in these patients. There is no consensus on whether there is any acceptable degree of haemolysis following a drug intervention for malaria in clinical cases or in asymptomatic individuals.

### Modeling the potential use of primaquine

**Figure 1:**
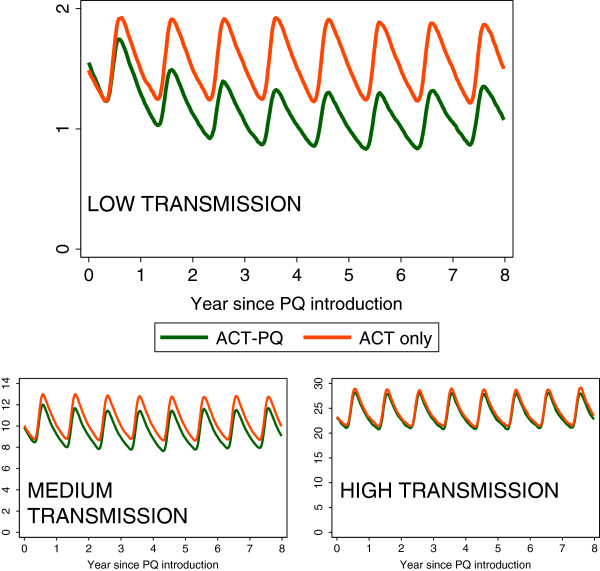
**Preliminary modeling of administration of primaquine together with ACTs in a range of transmission settings.** A simulation of adding primaquine to ACT first line treatment versus ACTs only in a seasonal setting. In this simulation 80% of clinical cases are treated with ACT alone or ACT-PQ. Primaquine is assumed to reduce the duration of infection by 78% and the level of infectiousness by 67% in treated patients compared to those treated with ACT alone. In a low transmission scenario, adding PQ to the treatment of clinical cases causes a higher relative reduction than in higher transmission scenarios. Ro differs between settings. Migration is not allowed for. With the kind permission of Lucy Okell, Jamie Griffin & Azra Ghani. For further details, see reference [40] .

**Figure 2:**
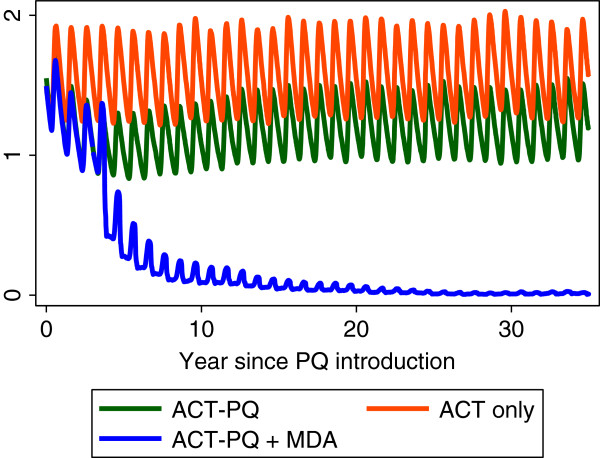
**The effect of annual MDA with primaquine in addition to primaquine and ACT treatment of clinical cases in a low transmission setting.** In a seasonal, low transmission setting, giving ACT+ primaquine to clinical cases plus an annual MDA with ACT plus primaquine could reduce malaria levels close to elimination if repeated for a number of years; however the model does not allow for immigration of malaria cases. With the kind permission of Lucy Okell, Jamie Griffin & Azra Ghani. For further details, see reference [40] .

Teun Bousema discussed how to extrapolate the effect of primaquine in the individual to community-level transmission, acknowledging that the infectious reservoir of malaria may vary with transmission setting. A recent model by Lucy Okell and colleagues [33] suggests that infectiousness post ACT alone is 13 days and post ACT plus primaquine is 3 days. Using this model that incorporates population age structure, immunity, heterogeneous exposure and as well multiple interventions as covariates, the addition of primaquine to ACT as first-line treatment significantly reduces transmission in low endemic settings but not in higher transmission settings (Figure 1). The proportion of people who received primaquine in addition to ACT is a key parameter suggesting primaquine needs to be given with all courses of ACT to have an effect. The models were further extended to investigate the effect of primaquine as part of an MDA [40] in a non-seasonal setting with 9% prevalence of *P. falciparum*. Giving MDA every four months caused an 80% reduction in transmission, but not elimination. With MDA every six weeks one could plausibly reach elimination. Preliminary models suggest that MDA may be more successful in areas of seasonal transmission (Figure 2). The duration of drug action is important and a long acting ACT plus a long acting 8-aminoquinoline could be an optimal combination.

An approach targeting malaria transmission hotspots may be appropriate for all endemic settings [8]. The hypothesis is that hot spots catalyse transmission and targeting them would reduce transmission both within and outside the hotspot. Modeling hotspot interventions with no drug treatment but with insecticide-treated bed nets scaled up to 80% coverage and targeted IRS had a significant effect on transmission. The effect of adding primaquine should be investigated. Models of transmission assume a long time-course and there was discussion as to the stability of hot spots and how this would affect the efficacy of an intervention.

## Meeting outputs

### Possible approaches for the use of primaquine to interrupt malaria transmission

**Figure 3:**
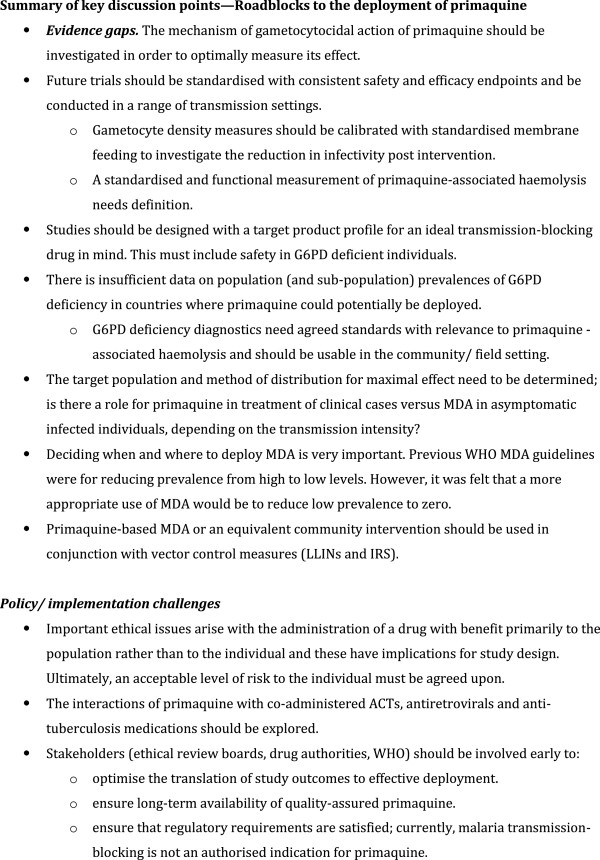
Summary of key discussion points—roadblocks to the deployment of primaquine.

**Figure 4:**
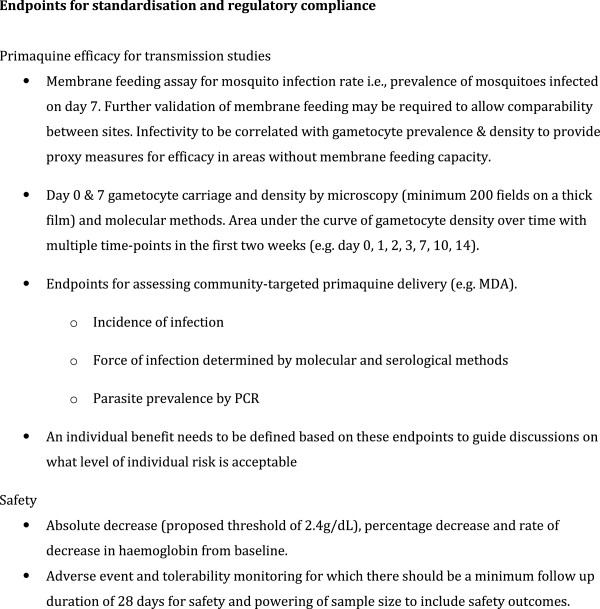
Endpoints for standardisation and regulatory compliance.

**Figure 5:**
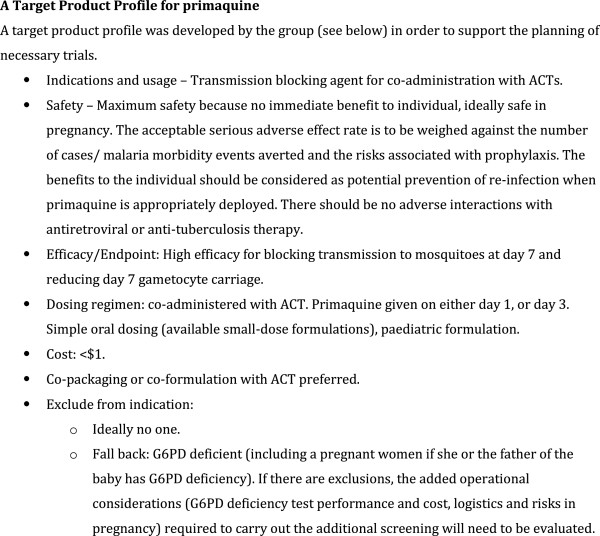
A Target Product Profile for primaquine.

**Figure 6:**
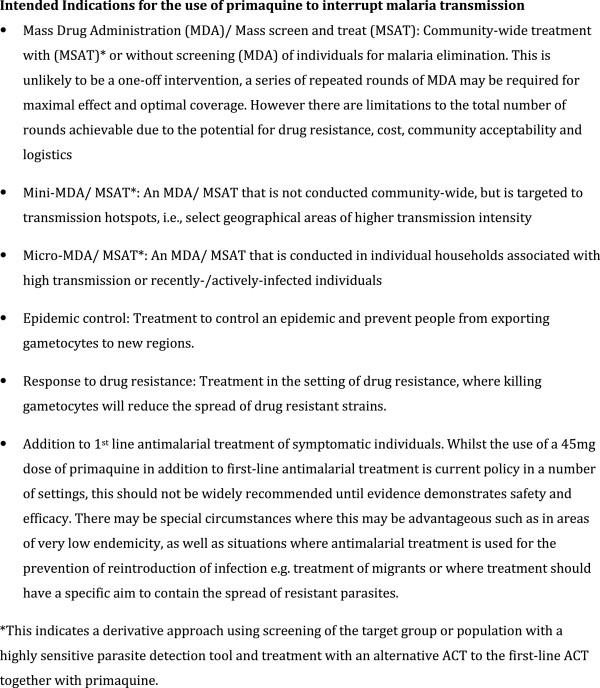
Intended indications for the use of primaquine to interrupt malaria transmission.

Having reviewed the existing data, the second aim of the meeting was to identify the roadblocks to deployment of primaquine in Africa (Figure 3), decide on common study endpoints and to determine the next steps. As a starting point the group determined the intended indications of primaquine (Figure 4), a target product profile (Figure 5) and common endpoints for infectivity, efficacy and safety studies (Figure 6).

## Key roadblocks to the deployment of primaquine

### Safety and efficacy of primaquine

The paucity of evidence for primaquine’s safety and efficacy for transmission-blocking were seen as major issues, particularly, the lack of data supporting the safest and most efficacious dose. If primaquine is going to be used to maximal benefit then it must be safe to deploy in G6PD deficient individuals and women of childbearing age and it must be safe to co-administer with HIV and tuberculosis treatments without adverse drug interactions. Most crucially, evidence is lacking for any transmission-reducing effect in the community from the addition of primaquine to routine anti-malarial treatment of symptomatic individuals.

### Suitable endemicity for use of primaquine

It was agreed that use of primaquine is most likely to have an impact on transmission intensity in areas characterized by low endemicity prior to the intervention, i.e. *P. falciparum* parasite rate (PfPR) by microscopy of less than 5%, or an EIR (entomological inoculation rate) less than 1. In such settings, there is a low frequency of symptomatic parasitaemia so the greatest benefit is likely to result from treating asymptomatic infections as well, through MDA or screen-and-treat initiatives. The optimal strategy for delivering primaquine-based MDA in terms of who to treat, at what threshold endemicity, with what regimen and how often is unknown.

Mathematical modelling indicates a limited effect at higher transmission intensities (PfPR> 10%). However, further iterations are needed to assess the additional effect of primaquine interventions together with other control tools at a range of transmission intensities. As was the situation in Aneityum, Vanuatu [41], there may be other higher transmission settings where interruption of transmission could occur using MDA with primaquine because of limited human migration.

### Partner drug for primaquine

For community campaigns with primaquine, the partner ACT should probably differ from the recommended first-line anti-malarial treatment. An alternative ACT may be required for community-wide MDA or in circumstances where repeated rounds of MDA are envisaged. However, in smaller hotspots of high transmission intensity where fewer rounds of treatment with ACT-primaquine are needed, the standard first-line ACT could be considered as the partner to primaquine. The relative gametocytocidal activity of the partner ACT, its half-life for killing asexual parasites and the potential for drug interactions or for synergy with primaquine will need to be considered.

### Drug supply and regulation

The manufacture and supply of the appropriate dose and formulation of primaquine was seen as a major obstacle for primaquine deployment. Currently, there are primaquine shortages globally and in Africa, the procurement of supplies to treat *P. vivax* where it is endemic is a challenge.

Further information on the current challenges for the manufacture and supply of single- or low-dose primaquine is required. A review of the current situation of primaquine manufacture and supply should be carried out with the aim of identifying the steps needed to ensure an adequate supply of primaquine formulated in the correct dose should low dose primaquine be found to be efficacious. It is likely that primaquine for the clearance of *P. falciparum* gametocytes will remain off label. In order to ensure the smooth process from manufacture to implementation, it was recommended that stakeholders from industry and governments, including regulatory authorities be brought together to discuss these challenges.

### Alternatives to primaquine

The meeting agreed that seeking alternative gametocytocidal drugs to primaquine was paramount due to the safety concerns with 8-aminoquinolines. The 8-aminoquinoline tafenoquine appears to have a similar safety profile to primaquine (haemolysis in people with G6PD deficiency), but being long-acting, may potentially inhibit gametocyte infectivity for longer. Should a safe, low dose be found, tafenoquine could be a useful tool in the elimination of *P. falciparum*. There is increasing evidence for methylene blue having a better safety profile [42,43], but more work needs to be done on regimen, dose-finding and acceptability [44,45]. The group supported the further development of these drugs and considers it a priority to develop more compounds active against transmission stages for all species of malaria.

### The roadmap

**Figure 7:**
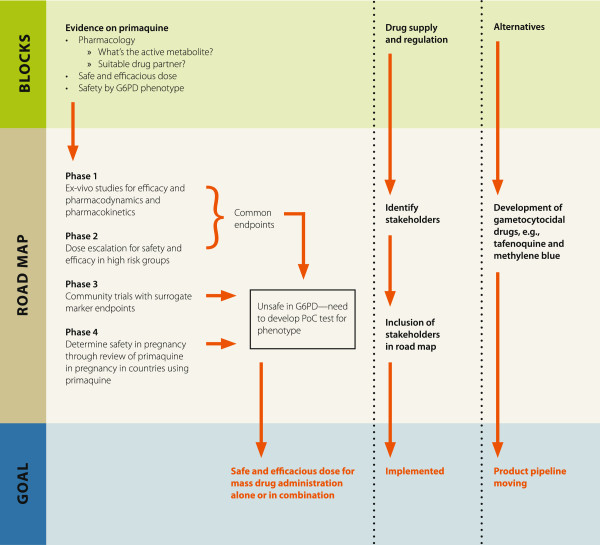
A projected roadmap for primaquine studies.

Three themes were identified that need to be addressed simultaneously. Firstly, there are evidence gaps for primaquine itself, secondly, the manufacture and supply of primaquine needs mapping and thirdly, efforts to search for a safe and effective alternative to primaquine need to be supported. A schematic of the roadmap is shown in Figure 7.

### Providing evidence of the efficacy and safety of primaquine

A range of studies from phase 1–4 were proposed that would inform decisions on the efficacy and safety of primaquine. These are outlined below.

#### Phase 1: Identification of the lowest dose for efficacy

*Ex vivo* gametocytocidal/ infectivity assays: because the active metabolites of primaquine are currently unknown, the interpretation of *in vitro* assays with primaquine is complicated. A possible approach would be to use healthy volunteers treated with different doses of primaquine. The plasma (containing primaquine metabolites) of these individuals could be used in membrane feeding experiments with cultured parasites to demonstrate lack of infectivity in mosquitoes of different doses of primaquine and in combination with ACT.

There is no proven relationship between mosquito infectivity and gametocytocidal effects so this may need to be repeated with a variety of parasite lines and volunteers of different ethnic backgrounds.

It was noted that much needed pharmacokinetic studies could be performed during the same experiments as could studies evaluating the different partner ACT, other gametocytocidal drugs and drugs in common use that may interact with primaquine (e.g. antiretrovirals and drugs for tuberculosis).

#### Phase 2: Establish the safety and efficacy of the optimal dose of primaquine in relevant sub-groups

Efficacy of low dose primaquine to assess post-treatment infectivity using common endpoints (see below) in G6PD normal individuals. A dose-finding study is currently under way in Uganda (NCT01365598).

Studies to confirm safety of low dose primaquine in G6PD deficient.

hemizygous males with lowest doses (dose escalation studies)

heterozygous females (dose escalation studies)

individuals of a given phenotypic G6PD enzyme function level, to establish a relationship between G6PD enzyme function level and safety, a proposed threshold enzyme function being in the range 20-30%.

Confirm safety and efficacy in infected population of unselected G6PD status (timeline 3–4 years).

If safety with G6PD deficiency remains a problem, field usable and reliable point of care tests to detect G6PD deficiency will be needed and the effect of not treating a proportion of the population on transmission reduction modeled.

Programmes to map the geographical distribution of G6PD deficiency in countries targeted for primaquine deployment. This should include assessment of the range of enzyme function levels in the population.

#### Phase 3: Studies to establish utility at community level

These may measure transmission reduction but may not necessarily need to be in the form of randomized controlled trials. Much can be learnt from the transmission-blocking vaccine field where designs such as a ‘stepped wedge’ design may be used with a focus on the indirect and community effects. Both prospective and retrospective pharmacovigilance studies will be needed and pregnancy registers will be an important component.

#### Phase 4: Studies to review the safety in pregnancy

Currently there is no evidence on safety of primaquine in pregnancy. Post-marketing surveillance is possible as several countries have adopted primaquine as policy, such as India, China and Sri Lanka. In these countries, pharmacovigilance could be supported to do a retrospective study following up women of reproductive age who have been treated with any dose of primaquine.

## Conclusion

Primaquine may be a useful malaria control tool in low-endemic settings in Africa when used in combination with a blood schizontocide. For maximal effect it will need to be given to asymptomatic parasite carriers and therefore a safe and efficacious dose needs to be found that can be used in populations with G6PD deficiency. Studies designed to find this dose should contain common endpoints including infectiousness to mosquitoes seven days after treatment and gametocyte prevalence pre-treatment and seven days post-treatment to allow maximal comparability between trials. Safety endpoints need to be defined, particularly with regard to G6PD pheno- and genotype and pregnancy. Methylene blue and tafenoquine are alternative drugs but need further testing and establishing standard protocols could facilitate this process. Community trials should identify the added benefit of using primaquine in addition to a long-acting ACT with the endpoint of community transmission reduction.

## Abbreviations

ACT: Artemisinin combination therapy; As: Artesunate; AUC: Area under the curve of gametocyte density over time; CQ: Chloroquine; EIR: Entomological inoculation rate; G6PD: Glucose-6-phosphate dehydrogenase; IRS: Indoor residual spraying; LLINs: Long-lasting insecticidal nets; MDA: Mass drug administration; PfPR: *P. falciparum* parasite prevalence; PQ: Primaquine; QT-NASBA: Quantitative real time nucleic acid sequence-based; SAE: Severe adverse event; SP: Sulphadoxine-pyrimethamine; WHO: World Health Organization.

## Competing interests

The authors declare that they have no competing interests.

## Authors’ contributions

ACE, RG and CD wrote and edited the manuscript, ACE, MH and JH were meeting rapporteurs and together with TB and LvS, contributed to the manuscript. All authors read and approved the final manuscript.
